# Differential diagnosis of CRMO

**DOI:** 10.1186/1546-0096-13-S1-P208

**Published:** 2015-09-28

**Authors:** A Kozlova, V Roshchin, D Abramov, N Bolshakov, A Roppelt, D Yuhacheva, A Shcherbina

**Affiliations:** 1 Federal Research and Clinical Center of Pediatric Hematology, Oncology and Immunology, Moscow, Russian Federation

## Introduction

Chronic recurrent multifocal osteomyelitis (CRMO) is a term, referred to a group of several autoinflammatory disorders (some of unknown genetic background) of children and young adults that is characterized by non-infectious osteomyelitis with or without high inflammatory activity and occasionally involvement of other organs. Patients typically present with bone pain secondary to multifocal osseous lesions, the disease has a remitting course. To specialists who care for patients with autoinflammatory disorders the clinical presentation of CRMO is very recognizable. Yet in the settings of multi-specialty clinic, a newly referred patient with bone lesions poses a certain diagnostic challenge and thorough differential diagnosis is required.

## Objectives

We conducted a study analyzing diagnosis and outcomes of children referred to tertiary center with bone lesions in 2014. Children with bacterial osteomyelitis were not included in the study.

## Methods

Laboratory, radiological tests, bone lesion biopsies were performed in all cases, other types of tests - as were required by clinical situation.

## Results

Most of the patients were diagnosed with various oncological/ oncohematological disease: Ewing's sarcoma was found in 22% of cases, osteosarcoma - 29%, histiocytosis X - 9%, bone metastases - 9%. In 24% of cases the disease was not associated with bone tissue: chondroblastoma, bone cyst, synovial sarcoma, osteochondroma etc. Only 5% of patients we were able to confidently diagnose chronic multifocal osteomyelitis

## Conclusion

In conclusion, newly diagnosed bone lesions in children require joint diagnostic efforts of various specialist. Our study showed, that in most cases swift and correct diagnosis and pathogenic treatment was only possible upon biopsy.

